# Supplementary material for: Dengue Virus Hijacks a Noncanonical Oxidoreductase Function of a Cellular Oligosaccharyltransferase Complex
Source: mBio. 2017 Jul 18;8(4):e00939-17. doi: 10.1128/mBio.00939-17 (PMC5516256; doi:10.1128/mBio.00939-17)
Supplement: TABLE S1 [file mbo004173397st1.docx]

**Table S1. Comparison of hits from the screen**

A list of the top 25 significant hits from biological replicates were compared to hits from two previously published screens. Gene names are colored to indicate groups of ER complexes. Yellow indicates genes encoding components of the EMC complex, blue indicates components of the OST complexes, and green indicates components of the ER associated degradation pathway (ERAD). The rank of each hit in independent biological replicates is shown in the second and third columns. The false discovery rate calculated by MAGeCK and the number of sgRNAs classified as a hit by MaGECK in our screen are shown. Hits from our screen that were also identified in the top 25 hits of Marceau *et al.* or the top 150 of Savidis *et al.* are marked with an X.

| ID | 1st rank | 2nd rank | 1st FDR | sgRNAs | Top 25 Marceau et al. | Top 150 Savidis et al. |
| --- | --- | --- | --- | --- | --- | --- |
| EMC6 | 1 | 2 | 0.00045 | 6 | x | x |
| STT3A | 2 | 11 | 0.00045 | 6 | x | x |
| MAGT1 | 3 | 5 | 0.00045 | 6 | x |  |
| STT3B | 4 | 3 | 0.00045 | 5 | x |  |
| UBE2J1 | 5 | 16 | 0.00045 | 6 |  |  |
| EMC3 | 6 | 12 | 0.00045 | 6 | x | x |
| EMC4 | 7 | 17 | 0.00045 | 6 | x | x |
| EMC5 | 8 | 4 | 0.00045 | 5 | x | x |
| EMC1 | 9 | 13 | 0.00045 | 6 | x | x |
| EMC2 | 10 | 7 | 0.00045 | 4 | x | x |
| RPN2 | 11 | 9 | 0.00045 | 5 | x | x |
| APOB | 12 | 10 | 0.002063 | 5 |  |  |
| SSR2 | 15 | 20 | 0.015161 | 4 | x |  |
| SSR3 | 16 | 14 | 0.015161 | 4 | x |  |
| HSPA13 | 17 | 15 | 0.033489 | 4 | x |  |
| OST4 | 18 | 18 | 0.058581 | 3 | x |  |
| PRKRA | 19 | 50 | 0.075 | 3 |  |  |
| UBE2G2 | 20 | 1 | 0.075 | 4 |  |  |
| SEC61B | 22 | 6 | 0.083483 | 5 |  | x |
| SSR4 | 25 | 48 | 0.121668 | 4 |  |  |
| SYVN1 | 26 | 8 | 0.121668 | 4 |  |  |
| RRBP1 | 30 | 21 | 0.21086 | 4 |  |  |
| OSTC (DC2) | 53 | 19 | 0.570164 | 3 | x |  |
| DERL2 | 74 | 84 | 0.768196 | 4 |  |  |
