# Supplementary figures and images for: Dengue Virus Hijacks a Noncanonical Oxidoreductase Function of a Cellular Oligosaccharyltransferase Complex
Source: mBio. 2017 Jul 18;8(4):e00939-17. doi: 10.1128/mBio.00939-17 (PMC5516256; doi:10.1128/mBio.00939-17)

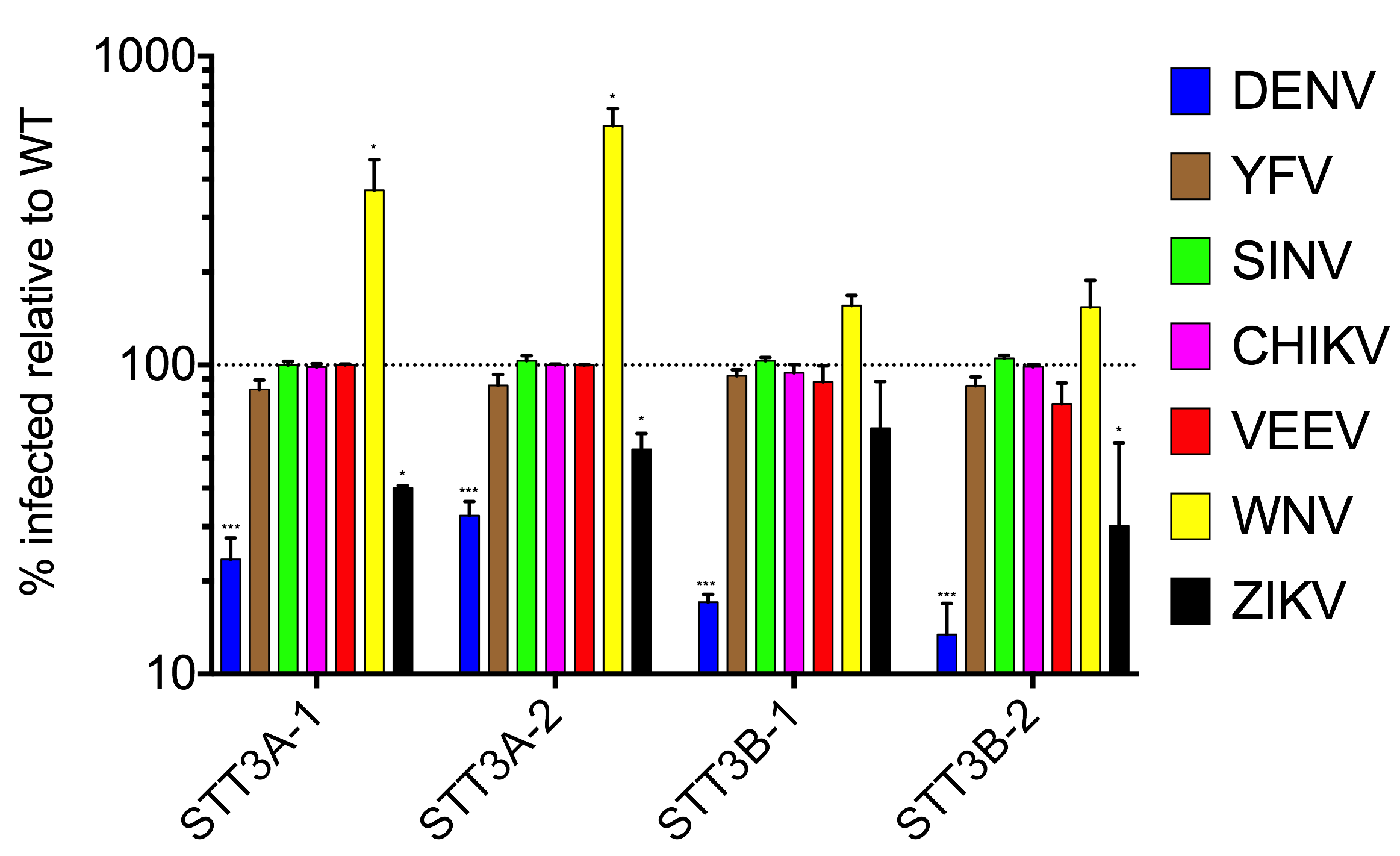

Supplement: FIG S1 [file mbo004173397sf1.tif]

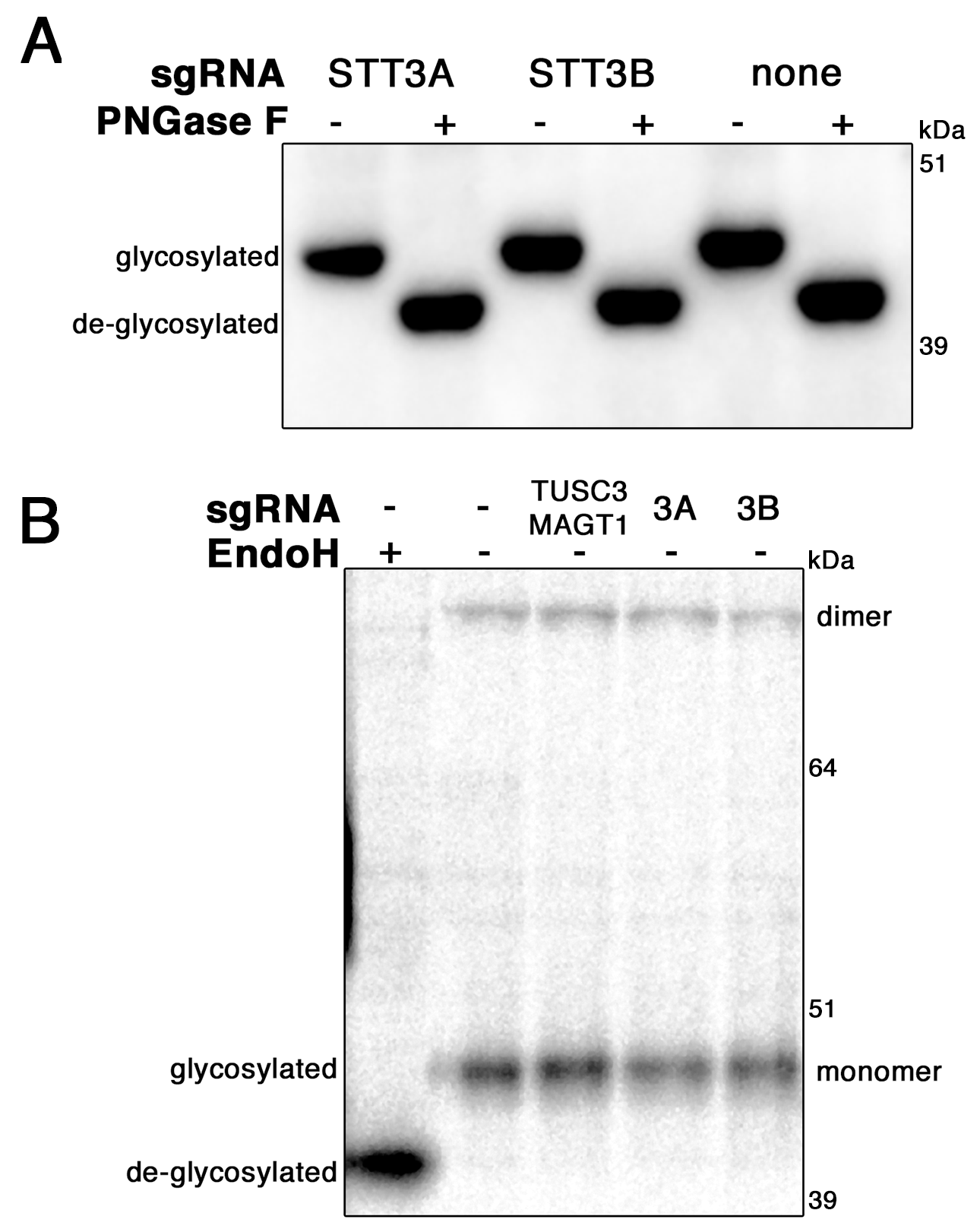

Supplement: FIG S2 [file mbo004173397sf2.tif]

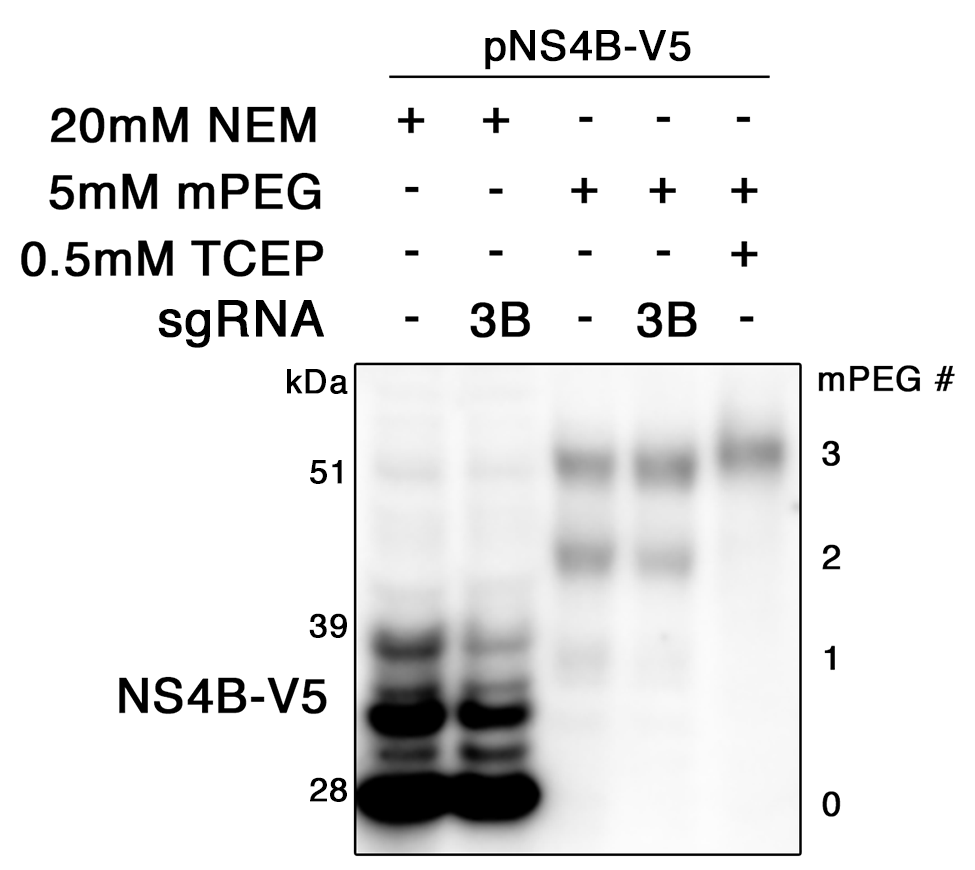

Supplement: FIG S3 [file mbo004173397sf3.tif]

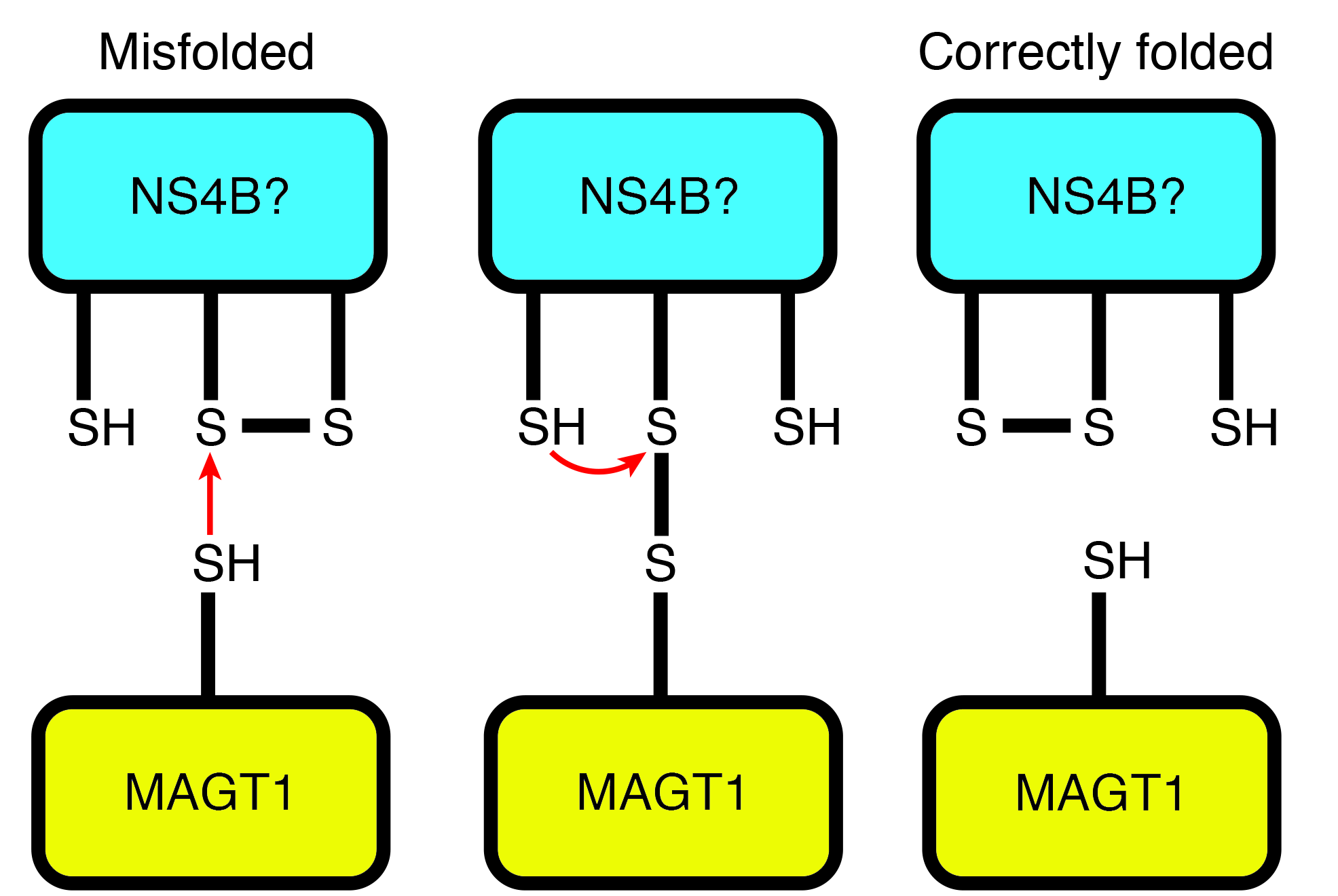

Supplement: FIG S4 [file mbo004173397sf4.tif]
