# Supplementary material for: Dengue Virus Hijacks a Noncanonical Oxidoreductase Function of a Cellular Oligosaccharyltransferase Complex
Source: mBio. 2017 Jul 18;8(4):e00939-17. doi: 10.1128/mBio.00939-17 (PMC5516256; doi:10.1128/mBio.00939-17)
Supplement: TABLE S2 [file mbo004173397st2.docx]

**Table S2. List of crRNAs used to generate knockout cells**

Oligonucleotides were cloned into pLENTICRISPRv2 to generate lentiviruses for CRISPR mediated knockout of specific OST genes.

| crRNA sequence | Comment |
| --- | --- |
| ACAGACATTCCGAATGTCGA | STT3A-1 |
| AAGGTGGTACGTGACGATGG | STT3A-2 |
| GATGTAAGGCCGCTAAAAGT | STT3B-1 |
| CCAGCGGTTATCATCAACCC | STT3B-2 |
| GAGCGAACATGGCAGCGCGT | MAGT1-1 Forward |
| AGCAGTGAACATGACGATAA | MAGT1-2 Forward |
| GGGCGAGGAGCTGTTCACCG | EGFP-1 from Addgene |
